# 结合课程特点开展课程思政的教学实践:以高效液相色谱法实验为例

**DOI:** 10.3724/SP.J.1123.2024.04008

**Published:** 2025-03-08

**Authors:** Huili YANG, Gangfeng TANG, Jie LEI

**Affiliations:** 复旦大学化学系, 上海 200433; Department of Chemistry, Fudan University, Shanghai 200433, China

**Keywords:** 课程特点, 课程思政, 仪器分析实验, 创新意识, 仪器设计思想, course characteristics, ideological and political education, instrumental analysis experiment, innovation awareness, instrument design philosophy

## Abstract

针对课程思政案例易重复,以及课程思政考评难的问题,本文以仪器分析实验中的经典实验高效液相色谱法为例,介绍了结合课程特点开展课程思政的教学实践。实验的具体内容为测定食品中的防腐剂和甜味剂;普适性和专属性两类课程思政案例的具体实践过程,包括案例意义、案例导入、案例实施、案例拓展和效果考核,普适性案例以仪器分析实验的重要性为例,专属性案例以高效液相色谱仪的国产化之路为例。此外,阐述了在仪器分析实验课程内,根据课程进度和学生实际情况,适时调整课程思政的内容,分阶段融入课程思政的重要性。除了价值引领和思想教育的功能,本文同时强化了课程思政在促进专业知识学习和创新能力培养方面的作用,并主张通过专业知识的掌握和创新能力的提高来评价课程思政的效果。

## Abstract (en)

In response to the problems of the easy repetition of ideological and political education cases and the difficulty of evaluating ideological and political education, this study takes the classic experimental method of high performance liquid chromatography as an example to carry out the teaching practice of ideological and political education based on the characteristics of the course. First, the aim of the experiment, that is, the determination of preservatives and sweeteners in food, was introduced. Next, the specific practice processes, including case significance, case introduction, case implementation, case expansion, and effectiveness assessment, of two types of ideological and political programs, namely, universal and specialized cases, were introduced. Universal cases consider the importance of instrumental analytical experiments, while specialized cases consider the localization of high performance liquid chromatographs. We adjusted the ideological and political content of the instrumental analytical experimental course according to the course progress and actual experience of students, and integrated ideological and political education into the curriculum in stages. In addition to the functions of value guidance and ideological education, the role of ideological and political programs in promoting professional knowledge learning and innovation ability training was strengthened. We recommend that the effectiveness of ideological and political education be evaluated by mastering professional knowledge and improving innovative abilities.

课程思政建设和研究至今已近十年^[1]^,其强调知识传授、能力培养与价值引领的同频共振^[1,2]^。化学类课程思政建设已从零星思政案例建设发展到课程体系的建设^[2]^。在目前分析化学类的课程思政建设研究中,更加注重课程思政的案例建设,同时发挥案例的价值引领和思想教育的功能^[3⇓⇓⇓⇓⇓⇓⇓-11]^,但对案例在促进专业知识学习和创新能力培养方面的强化不够。复旦大学分析化学类课程已开展课程思政多年,自2019年参与复旦大学第三批课程思政示范课程建设以来,已从之前侧重深挖课程相关的思政案例,逐渐转为系统、深入开展课程体系的思政实践和探索,在分析化学实验课程教学中引入模拟创业^[12]^;在理论课教学中引入病毒及其检测方法^[13]^,从而培养学生的创新创业意识和环保意识等,并侧重强调课程思政在促进专业知识学习和创新能力培养方面的作用。

仪器分析实验是化学类本科人才培养体系中的专业核心课程。由于仪器数量的限制等因素,实验教学安排均采用循环式,也就是教师在一学期内指导两个固定的实验,学生会在不同实验之间循环,因此实验指导教师每周接触到的学生均有不同学情。我们在进行课程思政教学设计时,充分结合该课程特点,循序渐进、分层次地开展课程思政,并将课程思政案例分为普适性和专属性案例两大类,课程前两周可以采用普适性的案例,以课程思政中的思想政治部分^[2]^为主,同时可引起学生对课程的兴趣和重视;课程后期则采用专属性的案例,以课程思政中的世界观、方法论和素质素养部分^[2]^为主。这样可避免案例的重复,同时可促进学生的专业知识学习和创新能力培养。

课程思政在实验课程教学中需要具体落实到单个实验的教学中。高效液相色谱法(high performance liquid chromatography, HPLC)实验在各高等学校普遍开设,该实验涉及样品前处理方法及仪器原理、仪器使用方法和仪器结构等方面。我们选取HPLC这一仪器分析典型方法,开展课程思政的教学实践示范。本文在前期工作^[12,13]^的基础上,以“高效液相色谱法测定食品中的防腐剂和甜味剂”为例,介绍仪器分析实验课程中开展普适性和专属性两类课程思政案例的具体实践过程。

## 1 实验部分简介

### 1.1 实验目的

能够说明高效液相色谱法的历史、基本原理和应用,以及高效液相色谱法分析的定性、外标法定量的方法;能够正确使用高效液相色谱仪,并能说明其结构、工作原理和设计思想;能够说明液相色谱柱的组成及常用填料的类型等。

### 1.2 内容提要

HPLC是现在色谱学的一个重要分支,其采用高压泵加快液体流动相的流速,采用微米级球形固定相以提高柱效。按固定相和流动相的极性不同,HPLC可分为正相色谱法和反相色谱法。反相色谱法中,固定相的极性小于流动相的极性,一般用非极性键合固定相;流动相为水或缓冲液,常加入甲醇和乙腈等与水互溶的有机溶剂以调节保留时间。反相色谱法适用于分离非极性和极性较弱的化合物,其应用范围比正相色谱法广泛得多。

防腐剂可以抑制食品中微生物的繁殖或杀灭微生物,保持食品的鲜度和良好品质,苯甲酸和山梨酸及其盐类都是常用食品防腐剂,糖精钠是食品中常用的人工甜味剂。随着人们对绿色食品的要求不断提高,这些食品添加剂的食用毒性一直为人们所关注。本实验采用反相色谱技术同时测定上述3种食品添加剂,利用保留时间定性,外标法定量。

### 1.3 主要仪器、试剂与材料

Agilent 1260 Infinity Ⅱ液相色谱系统(美国Agilent公司); SHB-ⅢS循环水式多用真空泵(上海道京仪器有限公司); SB-5200D超声波清洗机(宁波新芝生物科技股份有限公司);尼龙针头式滤器(0.22 μm, 13 mm)、尼龙微孔滤膜(0.45 μm, 50 mm)(Titan公司)。

甲醇(色谱纯); 0.02 mol/L、2.0 mol/L乙酸铵溶液;1 mg/mL苯甲酸标准储备液;1 mg/mL山梨酸标准储备液;1 mg/mL糖精钠标准储备液;Carrez试剂Ⅰ:取15 g K_4_[Fe(CN)_6_] ·3H_2_O,用水溶解并稀释至100 mL; Carrez试剂Ⅱ:取30 g ZnSO_4_·7H_2_O,用水溶解并稀释至100 mL; 10%氨水溶液。实验溶液所用化学试剂均为分析纯,购自国药集团化学试剂有限公司;所用的超纯水均由Milli-Q系统(Millipore,美国)制备。

样品Ⅰ:碳酸型饮料,如雪碧、美年达、可乐等;样品Ⅱ:酒,如葡萄酒、果酒等;样品Ⅲ:乳制品,如酸牛奶、调味牛奶等。以上样品均为市售。

### 1.4 实验步骤

#### 1.4.1 样品溶液的制备

样品Ⅰ:取30~40 mL液体样品于50 mL干燥的烧杯中,超声脱气10 min,准确吸取5 mL于50 mL容量瓶中,加入适量超纯水,加入1.0 mL 2.0 mol/L乙酸铵溶液,加入Carrez试剂Ⅰ、Ⅱ各1.0 mL,加入超纯水至刻度、混匀,静置10 min。

样品Ⅱ:因样品中不含CO_2_气体,省略超声脱气步骤,其他步骤与样品Ⅰ脱气后的处理方法相同。

样品Ⅲ:如样品呈酸性,先用10%氨水溶液调至中性(用pH试纸检验),其他步骤与样品Ⅰ脱气后的处理方法相同。

#### 1.4.2 标准溶液的配制

定性溶液的配制:分别将苯甲酸、山梨酸和糖精钠的3种标准储备液用0.02 mol/L乙酸铵溶液稀释100倍。

混合标准溶液(100 mg/L)的配制:准确移取苯甲酸、山梨酸和糖精钠标准储备液(1 mg/mL)各10 mL于100 mL容量瓶中,用0.02 mol/L乙酸铵溶液稀释至刻度。

混合系列标准溶液的配制:分别准确移取以上混合标准溶液1、2、3、4、5 mL于5个25 mL容量瓶中,用0.02 mol/L乙酸铵溶液定容至刻度。

标准加入法工作溶液的配制:在4个50 mL容量瓶中,各准确加入5 mL经过前处理的样品溶液,然后依次加入0、1.0、3.0及5.0 mL混合标准溶液(100 mg/L),各加入2.0 mol/L乙酸铵溶液1.0 mL, Carrez试剂Ⅰ、Ⅱ各1.0 mL,加水至刻度、混匀,静置10 min。

#### 1.4.3 色谱条件

色谱柱:Agilent Eclipse XDB-C18 (150 mm×4.6 mm, 5 μm);流动相:甲醇-乙酸铵溶液(0.02 mol/L)(7∶93, v/v);流量:1 mL/min;柱温:25 ℃;检测波长:205 nm;进样体积:20 μL。仪器操作步骤参见实验室操作面板。

#### 1.4.4 测定

进样分析前,所有溶液都需用一次性针筒吸取上层清液并经0.22 μm针头式滤器过滤。用50 μL液相色谱进样针吸取50 μL上述试样,在六通阀“Load”位置推针后,扳动手动进样阀至“Inject”位置,即开始数据采集。依次将下列样品按上述方法进样:①苯甲酸、山梨酸和糖精钠的定性溶液;②系列混合标准溶液;③样品溶液;④标准加入法工作溶液。系列混合标准溶液和样品溶液的色谱图见图1。

**图1:**
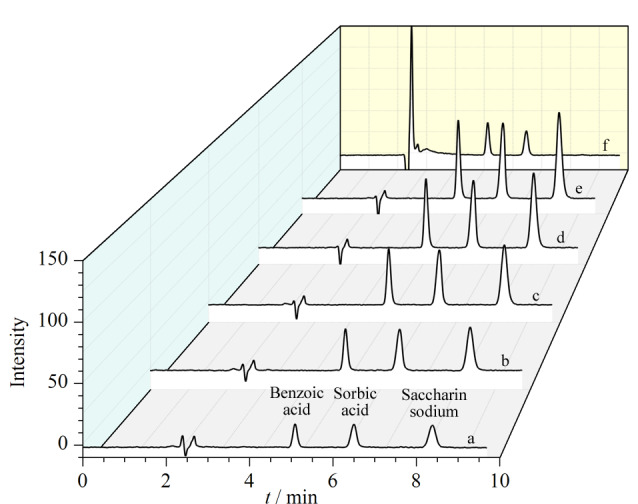
标准溶液和样品溶液的色谱图

## 2 课程思政案例的设计与实施

### 2.1 案例意义

本案例将高效液相色谱法应用于食品中防腐剂和甜味剂的测定,通过高效液相色谱法在食品安全领域的具体应用,使得学生了解分析化学的重要性,切身体会到分析化学可以使人们的生活更加安全、更加美好,同时进一步激发学生的社会责任感等。通过仪器检出限、食品添加剂使用标准中最大使用量等的比较,使得学生掌握分析化学中“量”的概念,并学会辩证地看待“食品添加剂”的安全问题。同时,在本实验课上强调实验成本和实验设计的重要性,设计和选择分析路线时既需要考虑实验效果,同时需考虑实验成本和环境污染等问题,以此培养学生的环保意识、大局意识等。此外,通过高效液相色谱仪的国产化之路,激发学生的创新意识和爱国热情,鼓励学生将所学与社会需求相结合。下面分别举例介绍两类案例。

### 2.2 普适性案例:仪器分析实验的重要性

#### 2.2.1 案例导入

根据每次课程学生不同的学情,由学生选取农业生产中的农药及其残留物的检验;工业生产中的原料分析、产品检验、生产工艺流程控制;社会生活中的兴奋剂检测、文物鉴定、刑事侦查等;与人们生活密切相关的食品检测、医学临床化验等方面的内容,了解科学技术研究的各个领域都需要应用分析化学相关实验技术解决具体问题。同时结合社会热点问题,比如食品安全问题(三聚氰胺事件、染色馒头问题等)、环境监测问题(企业污染物排放问题等)、病毒检测问题(冠状病毒的荧光检测法等),具体介绍分析化学方法是如何参与其中的。

#### 2.2.2 案例实施

前期安排学生通过自主学习,认识食品安全是一个综合概念,是企业和政府对社会最基本的责任和必须做出的承诺;课堂上组织学生介绍食品安全中令人关注的防腐剂和甜味剂的作用、含量及其常用检测方法。

高效液相色谱仪是食品安全检测的最常用仪器之一,实验前,与学生一起深入学习高效液相色谱法的实验原理、过程和特性,探讨将其用于检测防腐剂和甜味剂的优缺点(与其他分析检测方法作对照)。在实验间隙,组织学生查阅相关文献,总结高效液相色谱仪器发展历程,使学生认识到分析化学应用技术的发展不仅仅与发表高档次的论文和研究者个人发展直接挂钩,更是同国家、人类利益息息相关,需要研究者有家国情怀和社会责任感。

#### 2.2.3 案例拓展

要求不同组学生结合环境监测(企业污染物排放问题等)方面的实际案例,通过查阅文献,设计高效液相色谱法应用在具体污染物检测的方案,提升学生的实验设计和分析能力。他山之石,可以攻玉,文献报道过的分析化学及实验相关的课程思政探索各具特色^[3⇓⇓⇓⇓⇓⇓⇓-11]^,均可在具体教学实践中有选择性地引入高效液相色谱法的实验中。

#### 2.2.4 效果考核

学生能够通过课程论文说明分析化学应用技术发展的重要性,并能够通过调研或查阅资料,综述高效液相色谱法在食品安全领域中的应用。

### 2.3 专属性案例:高效液相色谱仪的国产化之路

#### 2.3.1 案例导入

实验科学均建立在测量的基础上,测量常常离不开仪器。据2021年科技日报报道,2018年的国家科技基础条件资源调查工作显示,在原值超过50万元的大型仪器中,国产仪器占有率仅为13.4%左右。到目前为止,部分高端分析仪器的核心技术仍然被国外控制,比如:冷冻电镜、核磁共振波谱仪和高端质谱仪等;此外,相应的专业技术人才也较为缺乏,仪器使用维护成本高,科研和教育经费大量外流。

液相色谱仪的国产化是中国几代色谱人的追求,我国液相色谱仪器的研制在20世纪70年代已经开始^[14]^,然而当时国产仪器在性能、性价比等方面均不如进口仪器,在市场上不具备竞争力。在此期间,各高校和科研单位研究人员研制和改进液相色谱仪器的经历是留给我们的宝贵精神财富^[15]^。结合学生前几周使用过的分析仪器,引导学生认识到仪器质量涉及方方面面,从设计思想、元器件、制造工艺到装配调试等,每一个环节都可能影响仪器的质量及其可靠性;研制、创新和生产仪器是非常有意义的工作,也具有很大的社会价值。

#### 2.3.2 案例实施

通过探究式学习,使学生了解高效液相色谱仪的历史、设计思想、仪器组成等;掌握高效液相色谱仪组成:溶剂输送系统、进样系统、分离系统、检测系统和数据处理与记录系统。高效液相色谱仪不同功能的部件,其设计制造都有很高的技术和精度要求,同时还要考虑不同部件的匹配问题和连接问题,从色谱系统可以学习整体和部分的辩证关系。例如本实验中欲将4.6 mm内径的色谱柱替换为内径小于100 μm的毛细管色谱柱时,就需要考虑进样装置、检测池和连接管路等匹配问题^[16]^,匹配度差会产生柱外效应导致色谱峰展宽;如果接头处管线长度稍短,则会形成死体积,导致色谱峰峰形畸变。

课堂间隙采用同伴互学,使学生明白高效液相色谱仪中关键的部件之一是液相色谱柱,其是由色谱填料、柱管、压帽、卡套、筛板、接头、螺丝等组成,每一个组成部件的质量都会影响到柱效。作为化学类专业的学生,需要辩证看待术业有专攻和全面发展的问题,找到自己专业匹配度最高的领域(色谱填料)进行深耕。色谱填料技术方面又包含基球的制备、键合工艺和装柱技术等,前两种技术更加需要化学知识,而装柱则是熟能生巧的技术。其中,关于基球的制备和键合工艺,引导学生具体分析文献报道过的色谱填料如果要商品化,需要攻克的关键问题是什么。此时学生会主动将理论课上所学的范氏方程等加以应用,理论指导实验,辩证地看待文献的优缺点,进而明白文献中的大量新型色谱填料为什么都没有产业化。最终引导学生深入了解企业研发部门和高校实验室研究思路的区别。

利用头脑风暴和课堂小测验,考查学生关于六通阀的设计思想。展示不耐高压的六通阀,使学生深刻认识到仪器设计思想和精密加工水平同样重要,工匠精神、创新思维缺一不可。

#### 2.3.3 案例拓展

引导学生对比不同专业类文献中HPLC相关的实验内容,总结仪器功能、性能和实验目的之间的联系,使学生明白“按需购买仪器”的可行性和必要性;大多时候,“购买最贵的仪器”不仅没有必要,反而会造成资源的浪费。

结合实验需求,引导学生创新设计纳升级的进样系统以及四通阀、十通阀等;让学生结合近年来文献报道过的新型材料,讨论将其开发为新型色谱柱填料的可行性。推荐学生课后阅读仪器分析方面研发、创业相关的书籍^[17]^,使学生认识到,创新创业需要全面的素质和能力,并不是专利加一腔热血即可。同时,鼓励学生整理、综述复旦大学自主研发科学仪器的故事^[18,19]^。

#### 2.3.4 效果考核

在课程小论文和读书笔记中,学生能够深刻阐述团队协作、学术诚信的重要性等^[17]^。在期末考试中,学生能够解释高效液相色谱仪各个组成部分的作用及关键部件;能够根据分离需求,选择、组装、设计不同的元器件及色谱仪器;能够通过调研或查阅资料,综述国产仪器的现状及发展方向等。

## 3 实施建议

高效液相色谱法测定食品中防腐剂和甜味剂的实验时长为8学时,在课堂前测内容中增加课程思政相关题目(约需增加5 min时间),了解每次实验的具体学情,结合学生的已有知识、兴趣和今后的人生规划等,选择1~2个案例开展课程思政教学。学期初的前两周,以能引起学生兴趣、激发学生学习热情的普适性思政案例为主。第三周开始,学生具备了仪器分析方面的基本知识,则可以开展专属性的思政案例教学,利用课程思政协助教学的过程中,需要精心安排课程内容,精心组织、合理穿插课程思政内容。课堂教学中的课程思政内容穿插于学生进样后的等待时间,不增加实验课时长。

目前,在分析化学教材及专著中有关仪器设计及研制的内容很少,为了能讲好、讲透这部分内容,还需要阅读大量的中外文献和总结发展历程。教育的目的是为了激发和引导学生自我发展之路,因此教学模式上,要实现从“以教为中心”向“以学为中心”转变,对教学方法、教学内容等进行再设计与实践,为学生创设高效的学习氛围。教学途径上,由于课程思政内容大都为描述性知识,因此建议将课程从课堂内延伸到课外,促使学生自觉开展深层次和成就式学习,并通过专业学习促进学生生涯意识的形成,提升专业学习的热情,树立学以致用的目标。

## 4 结语

课程思政实质是一种课程观,不是增开一门课,也不是增设一项活动,而是将课程思政融入课程教学的各环节、各方面^[1]^。由于学生的时间有限、视野不够开阔,这就需要实验课指导教师在实验过程中持续引导学生。本文结合仪器分析实验课程的特点,在课程内适时调整课程思政的内容,分阶段融入课程思政,并以经典实验高效液相色谱法为例,分别举例介绍了普适性和专属性课程思政案例的具体实践过程,以期实现以价值引领促进知识传授和能力培养。思想引领和价值引导是一个长期过程,是教师言传身教、所有课程协同作用的结果,几乎没法做到在一门课程内进行考评;如果从促进专业知识学习和能力培养的角度来考虑,则可通过专业知识的掌握和综合素质的提高来评价课程思政的效果,促进学生的全面成长成才。
